# Effect of electrolyzed high-pH alkaline water on blood viscosity in healthy adults

**DOI:** 10.1186/s12970-016-0153-8

**Published:** 2016-11-28

**Authors:** Joseph Weidman, Ralph E. Holsworth, Bradley Brossman, Daniel J. Cho, John St.Cyr, Gregory Fridman

**Affiliations:** 1 Thomas Jefferson University, Philadelphia, PA USA; 2 Southeast Colorado Hospital, Springfield, CO USA; 3 Independent Statistical Consultant, Conshohocken, PA USA; 4 Rheovector LLC, King of Prussia, PA USA; 5 Jacqmar, Inc., 10965 53rd Ave. No., Minneapolis, MN 55442 USA; 6 A. J. Drexel Plasma Institute, Camden, NJ USA

**Keywords:** Drinking water, Rehydration solutions, Fluid therapy, Human physical conditioning, Blood viscosity

## Abstract

**Background:**

Previous research has shown fluid replacement beverages ingested after exercise can affect hydration biomarkers. No specific hydration marker is universally accepted as an ideal rehydration parameter following strenuous exercise. Currently, changes in body mass are used as a parameter during post-exercise hydration. Additional parameters are needed to fully appreciate and better understand rehydration following strenuous exercise. This randomized, double-blind, parallel-arm trial assessed the effect of high-pH water on four biomarkers after exercise-induced dehydration.

**Methods:**

One hundred healthy adults (50 M/50 F, 31 ± 6 years of age) were enrolled at a single clinical research center in Camden, NJ and completed this study with no adverse events. All individuals exercised in a warm environment (30 °C, 70% relative humidity) until their weight was reduced by a normally accepted level of 2.0 ± 0.2% due to perspiration, reflecting the effects of exercise in producing mild dehydration. Participants were randomized to rehydrate with an electrolyzed, high-pH (alkaline) water or standard water of equal volume (2% body weight) and assessed for an additional 2-h recovery period following exercise in order to assess any potential variations in measured parameters. The following biomarkers were assessed at baseline and during their recovery period: blood viscosity at high and low shear rates, plasma osmolality, bioimpedance, and body mass, as well as monitoring vital signs. Furthermore, a mixed model analysis was performed for additional validation.

**Results:**

After exercise-induced dehydration, consumption of the electrolyzed, high-pH water reduced high-shear viscosity by an average of 6.30% compared to 3.36% with standard purified water (*p* = 0.03). Other measured biomarkers (plasma osmolality, bioimpedance, and body mass change) revealed no significant difference between the two types of water for rehydration. However, a mixed model analysis validated the effect of high-pH water on high-shear viscosity when compared to standard purified water (*p* = 0.0213) after controlling for covariates such as age and baseline values.

**Conclusions:**

A significant difference in whole blood viscosity was detected in this study when assessing a high-pH, electrolyte water versus an acceptable standard purified water during the recovery phase following strenuous exercise-induced dehydration.

## Background

Water is an essential nutrient for life, and hydration plays a critical role in human physical performance as well as in the prevention of chronic diseases. Dehydration is a well-accepted contributor to impaired human physical performance, resulting in guidelines established for fluid replacement in many professions involving significant physical activity, including athletes [1]. Performance impairments that are mediated by dehydration can produce untoward effects such as cardiovascular strain, heat strain, altered neurologic function and altered metabolic function [2].

Reductions in body mass by 2% or more due to perspiration during exercise have been well-established to be linked with impaired aerobic and physiologic performance. While this impairment involves metabolic, neurological, cardiovascular and important thermoregulatory factors, the primary limiting factor of exercise performance is cardiovascular drift, reflecting a shrinking cardiovascular reserve by reduced stroke volume and mean arterial pressure during intense or protracted exercise, coupled with an increase in heart rate [3]. Exercise-induced elevations in heart rate with a decrease in myocardial stroke volume can correlate closely with the degree of dehydration [2]. Dehydration has been shown to increase systemic vascular resistance by 17 ± 6% compared with euhydration during prolonged exercise (*p* < 0.05) [4].

Numerous studies have evaluated beverage rehydration around exercise sessions, which have included supplementation with water, coconut water, juices, teas, sodas, as well as carbohydrate, electrolyte and glycerol beverages [5–9]. In a majority of these studies, fluid replacement beverages were administered orally after a dehydration challenge and the rehydration abilities of specific replacement beverages were assessed using biomarkers, physical performance evaluations and subjective questionnaires. One study involving 6 healthy males suggested that higher vs. lower concentrations of a carbohydrate-electrolyte solution were more effective in restoring hydration following exercise [5]. A study of 10 soccer players reported that exercise-induced changes in body mass and plasma volume were smaller with the ingestion of a carbohydrate-glycerol beverage than a carbohydrate beverage, highlighting improved hydration with the addition of glycerol [6]. Another study which monitored hydration biomarkers showed that coconut water did not hydrate significantly better than water alone [7]. Alkaline water (ALK) has been hypothesized to be superior to standard purified water in restoring rehydration and high-shear blood viscosity during a 2-h recovery period following exercise-induced dehydration; however, specific structured studies of one or multiple biomarkers during re-hydration following exercise have not established a gold standard biomarker for recovery period. Therefore, we designed a randomized, double-blind, parallel arm research study to characterize and compare the magnitude and rate of rehydration of high-pH electrolyzed water vs. standard purified water by assessing serial levels of a specific biomarker of whole blood viscosity at high-shear rate as a primary endpoint. In addition to measuring whole blood viscosity at high shear rate, the following secondary endpoints were assessed: low-shear blood viscosity, plasma osmolality, bioimpedance, and changes in body weight.

## Methods

This study, performed at the Waterfront Technology Center (Camden, NJ), was a randomized, double-blind, parallel-arm, controlled trial, which recruited 100 adult volunteers (50 male, 50 female), between 25 to 49 years of age. Eligible participants were healthy, non-smoking adults, having a body-mass index less of 29 or less and free from any medication for at least one week prior to the participation in the study. Female participants were excluded from the study if they were pregnant, breast-feeding, menstruating at the time of screening, or if they had taken oral contraceptives in the previous 3 months. Subjects were instructed to refrain from strenuous activity, alcohol, and to limit excessive caffeine intake (>2 six-ounce cups) for at least 24 h prior to their assigned arrival on the study date. This clinical study was approved by the Institutional Review Board, and written informed consent was obtained from all subjects at the time of enrollment and prior to participating in this study. The study was registered (ClinicalTrials.gov Identifier: NCT02118883) and conducted in accordance and compliance with Good Clinical Practice and the Declaration of Helsinki.

### Design of study

The two different fluid replacement beverages consisted of standard bottled water as the control (CON), having a normal pH with minerals added for taste (Dasani®, The Coca-Cola Company, Atlanta, GA). The electrolyzed, high-pH ALK with added minerals for taste acted as the experimental treatment beverage (Essentia®, Essentia Water, LLC, Bothell, WA). Supplies of both water samples were stored in the same climate-controlled indoor location and covered to prevent prolonged light exposure.

Subjects were permitted to consume food and water at will prior to the study. Following a baseline assessment, participants were asked to refrain from food or fluid intake. Baseline assessments for body mass, bioelectrical impedance and vital signs (heart rate (HR), systolic (SBP) and diastolic blood pressure (DBP), respiration rate, body temperature) were collected at the initiation of the study prior to exercise. Blood samples were collected by venipuncture for evaluation of whole blood viscosity and plasma osmolality. Following baseline measures, the subjects performed moderate aerobic exercise sessions (using their choice of a treadmill, stationary bicycle, and/or elliptical trainer) in a warm environment (30 °C, 70% relative humidity) until they reached a dehydrated state. The duration of exercise varied between subjects; however, the dehydration threshold target was standardized to 2.0 ± 0.2% body weight loss due to the effects of a period of exercise in producing mild dehydration. During the exercise period, participants dried themselves thoroughly before each body mass measurement. A disposable paper gown of known weight was provided during body mass measurements. After the exercise period was completed and a dehydrated state attained, study participants moved to a thermo-neutral environment (21 °C, 60% relative humidity), where they rested for 20 min. After this rest period, vital signs, weight and bioimpedance were assessed. In addition, blood samples were collected for assessment of blood viscosity and plasma osmolality.

A prior study, assessing the effect of oral carbohydrate solution on rates of absorption reported an approximate 3% reduction in plasma volume during a 105-min interval after beverage consumption [10]. The present study incorporated a follow-up period of 120 min, which was considered to be sufficiently long in duration to show any effect of rehydration during recovery. The 120-min follow-up period (T000 to T120 min), which followed exercise and rest, was divided into a 30-min rehydration period and a 90-min recovery period. Participants were rehydrated orally by CON or ALK (T000 to T030 min). The mass of the water consumed during the rehydration period was calculated according to a participant’s body mass change during the exercise period. The recommended amount of rehydration fluids was determined using a formula of 20 mL of oral hydration per 1 kg of subject body weight, i.e. 2% of pre-exercise, baseline body weight. Water volumes poured into containers were measured using a precision scale (Intelligent-Lab PD-3000, Intelligent Weighing Technology, Inc. Camarillo, CA) by an unblinded coordinator who had no contact with any participants or study results throughout the study. Subjects were required to consume the entire quantity of designated water following exercise ad libitum within 30 min (T000 to T030 min). Blood samples were collected for whole blood viscosity and plasma osmolality at T015 min and T030 min during this rehydration period.

**Fig. 1:**
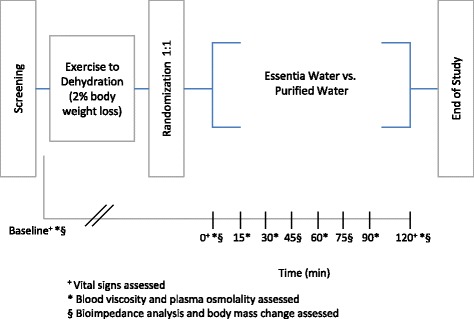
Study overview (clinical study flow sheet)

Additional data were collected during the 90-min recovery period (T030 to T120 min) to fully assess any potential variations in measured parameters. Blood viscosity and plasma osmolality were assessed seven times: at baseline and at six subsequent intervals (T000, T015, T030, T060, T090, and T120 min). Bioimpedance analysis and body mass change measurements were performed five times: at baseline and at four subsequent intervals (T000, T045, T075, and T120 min). Vital signs were evaluated a total of three times: at baseline, as well as at T000 and T120 min. A flow sheet showing time points for each biomarker evaluation is represented in Fig. 1.

### Measured parameters

#### Whole blood viscosity

Whole blood viscosity, the inherent resistance of blood to flow, was used as a measurement of intravascular hydration status. Blood viscosity was assessed across a physiologic range of shear rates of 1-1000 s^-1^ in increments of 0.1 s^-1^ using an automated scanning capillary tube viscometer (Hemathix SCV-200, Health Onvector, King of Prussia, PA). This instrument has been validated using rotating cone-and-plate and couette type viscometers across a range of shear rates [11]. Approximately 3 cc of whole blood were collected for each blood viscosity test. Each blood sample was processed and analyzed at 37 °C within 24 h after being collected. Blood viscosity levels were reported in millipoise units (1 centipoise [cP] = 1 millipascal-seconds [mPa•s] = 10 millipoises [mP]). Blood viscosity values, measured at a high shear rate of 300 s^-1^, were reported as systolic blood viscosity, and those measured at a low shear rate of 5 s^-1^ were reported as diastolic blood viscosity.

### Plasma osmolality

Once retrieving a blood sample, the plasma osmolality was assessed within 24 h. Each sample was centrifuged at 5 °C for 10 min at 1000 x *g*, and the plasma component was shipped to a reference laboratory (Laboratory Corporation of America, Burlington, NC), which performed the analysis using a freezing-point depression osmometer (Advanced Instruments, Norwood, MA).

### Bioelectrical impedance

Bioelectrical impedance analysis, or bioimpedance, was performed on site using a bioimpedance analyzer (Quantum IV, RJL Systems, Clinton, MI). Subjects assumed a supine position with their arms 30° from the body and their legs not touching. Electrodes were placed on the right hand and right foot of each subject and removed after each measurement. On the subject’s hand, the signal electrode was placed on the skin of the metacarpophalangeal joint of the middle finger, and the detecting electrode was placed on skin of the wrist. On the foot, the signal electrode was placed on the skin at the base of the second toe, and the detecting electrode was placed on the skin at the top of the ankle. The following indices were recorded during each measurement: impedance, reactance, capacitance, phase angle, total body water, intracellular water, and extracellular water.

### Body mass

Body mass index (BMI) was measured using a digital floor scale (HealthOMeter 349KLX, Pelstar, LLC, McCook, IL). Measurements were performed using a nude, dry weight, with a dry gown of known weight provided for comfort.

### Determination of sample size

The scanning capillary viscometer used to assess the primary endpoint in this study was previously employed in a preliminary study of dehydration and rehydration by high-pH alkaline water in 15 nonsmoking, apparently healthy firefighters. The variability of systolic blood viscosity measurements (high-shear viscosity) and the rehydration effect of high-pH alkaline on systolic viscosity observed in this prior study population were used to determine the sample size for this study [12]. In this firefighter trial, dehydration induced by fighting mock fires in training session with full equipment produced mean systolic viscosity values of 42.7 mP, and after rehydration, mean systolic blood viscosity was significantly reduced to 38.8 mP (*p* = 0.003). A standard deviation of 2.6 mP observed at baseline was used in determining our sample size for the present study. We postulated that high-pH ALK would demonstrate 40% greater rehydration effect than CON, that is, rehydration by CON was hypothesized to reduce mean systolic blood viscosity to 40.5 mP while ALK was hypothesized to reduce systolic blood viscosity to 38.8 mP from a dehydrated level of 42.7 mP. The present study was powered to detect such a contrast with 90% power using a type I error rate of 5%. This required 100 participants or 50 in the CON group and 50 individuals in the ALK group.

### Statistical analyses

Statistical analyses were performed using SAS (Statistical Analysis System, Version 9.3, 2012, Cary, NC). The data were analyzed using both descriptive and inferential statistics. Four separate analyses were pre-planned: comparison of percent change in biomarkers, comparison of the slopes of regression lines, absolute differences, and mixed model analyses.

A comparison of the percentage change of each outcome measure was performed during the rehydration and recovery period. Such an analysis was intended to compensate for the individual differences at baseline and at T000 min values. For example, the percentage change in the endpoint parameter from T000 to T120 was computed for whole blood viscosity (WBV) as:

$$ \frac{\mathrm{WBV}\left(\mathrm{T}000\right)\ \hbox{--}\ \mathrm{W}\mathrm{B}\mathrm{V}\left(\mathrm{T}120\right)}{\mathrm{WBV}\left(\mathrm{T}000\right)} $$

Mean values for each treatment group and estimates of standard errors for each enabled confidence intervals were to be computed and conclusions made based on these differences.

Fitting a line to each set of endpoint data for each variable, CON versus ALK were examined and statistical tests were conducted on the difference of the slope parameter for each line to determine if there was a significant overall treatment effect on the rate of rehydration during the recovery period. Regression procedure (PROC REG) was used in SAS to provide estimates of the best fitting line and of the slope and intercept parameters and to generate the data plots. Faster rehydration would be demonstrated for the group having a steeper slope for the line fit to the data between T000 and T120 min.

Absolute changes between baseline and each subsequent time point were also computed for each of the outcome parameters. Keeping the two assigned treatment groups separate, a plot of the mean values was performed for each of the outcome parameters at each time point starting at baseline and continuing through T120 min after commencing rehydration. By graphing each of the endpoints (y-axis) vs. time (x-axis), an initial change in the outcome measure between baseline and T000 was expected, as the latter was at or near the maximum point of dehydration and thus an expected inflection point of the endpoint parameters. Subsequently, a gradual restoration in these measures was expected as rehydration occurred. The mean value at T000 was expected to serve to indicate the dehydration level for each group. Mean and standard errors for each time point were to be computed, allowing tests at any particular time point to be made comparing the two treatment groups. Structuring 95% confidence intervals (using mean ± 1.96 S.E.) around each point enabled differences to be tested at every time point.

A final pre-planned analysis was employed for validation using a linear model approach but allowing for repeated measures generated for the outcome variables at all time points. In this analysis, a mixed model was used to specify observations at the different time points as random effects, and included fixed effects such as treatment (i.e., ALK vs. CON), age, baseline levels, and weight loss at end of exercise (%) in the analysis. Then, the treatment effect was estimated while controlling for these covariates. Using mixed model procedure (PROC MIXED) in SAS, the treatment effect comparing ALK vs. CON was tested for each of the outcome variables.

**Fig. 2:**
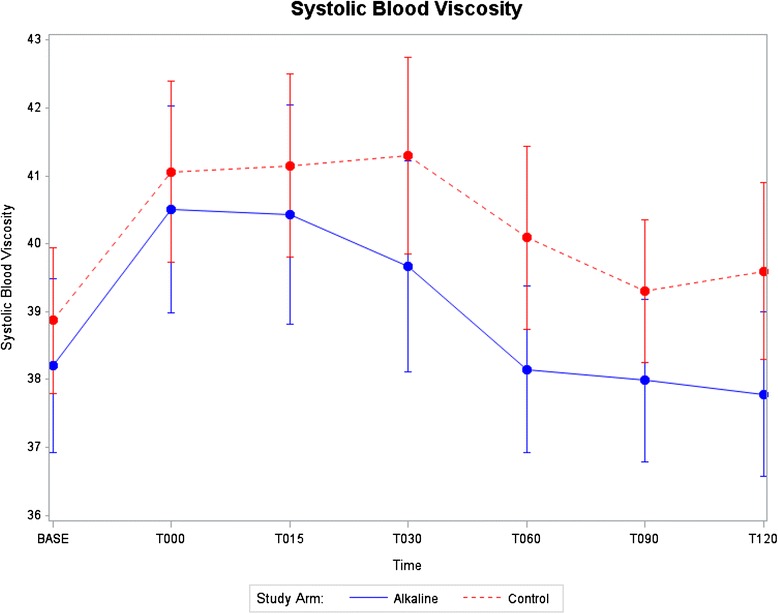
Systolic blood viscosity as a function of time for CON and ALK

**Fig. 3:**
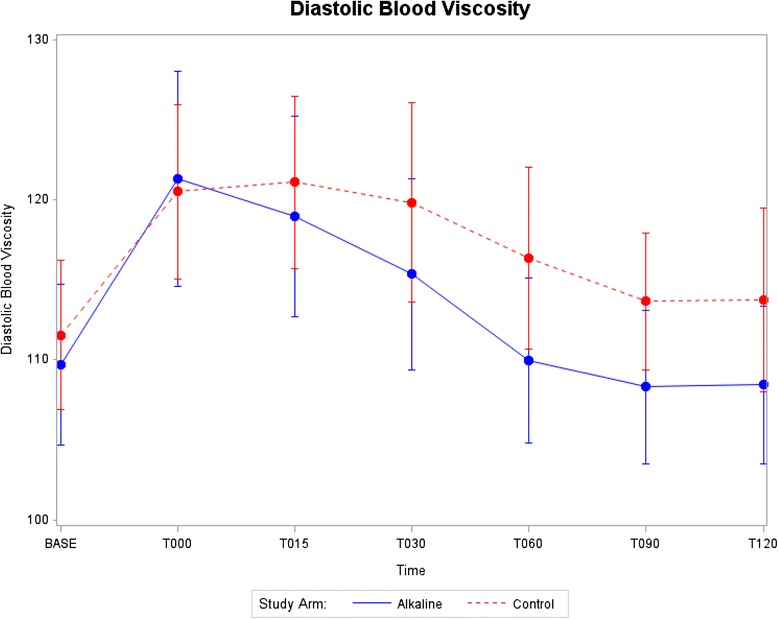
Diastolic blood viscosity as a function of time for CON and ALK

**Fig. 4:**
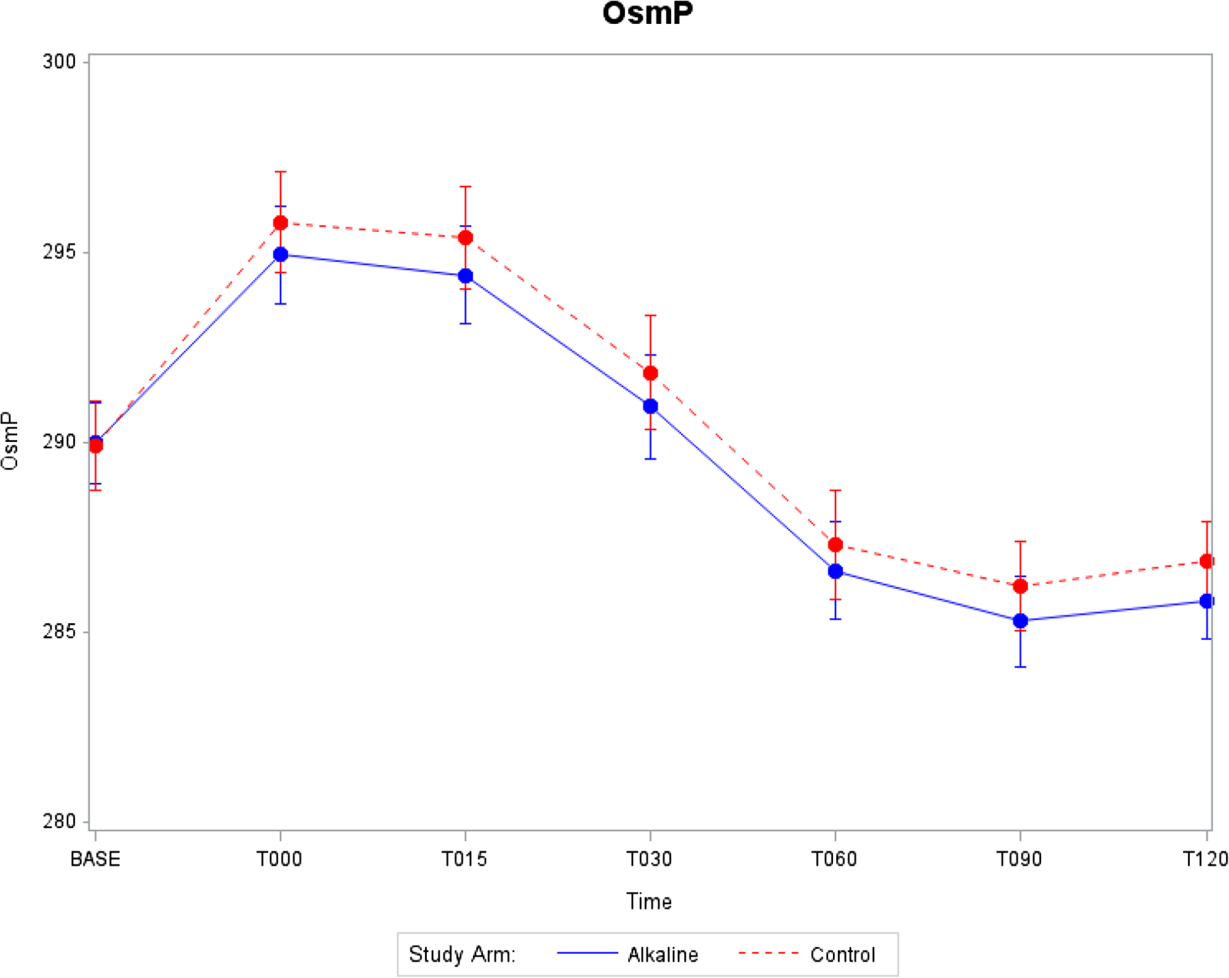
Plasma osmolality as a function of time for CON and ALK

**Fig. 5:**
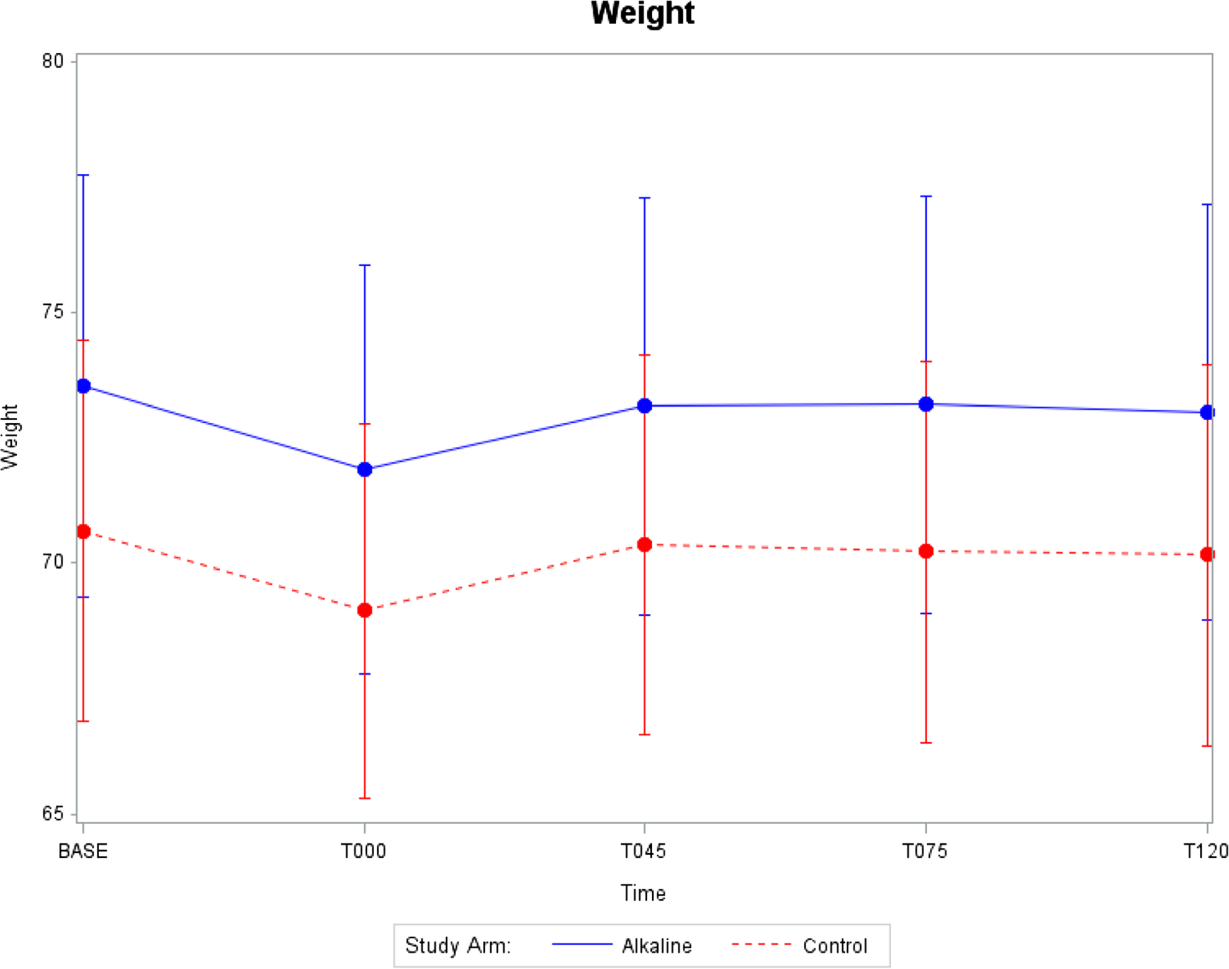
Body weight as a function of time for CON and ALK

Data displays of key outcome variables at each time point starting at baseline and continuing through T120 min after start of rehydration are provided in Figs. 2, 3, 4 and 5. Mean and standard errors for each time point were computed, allowing tests at any particular time point to be made comparing the two groups. Structuring 95% confidence intervals using mean ± 1.96 S.E. enabled absolute differences to be tested. As shown in Figs. 2, 3, 4 and 5, the 95% confidence intervals are displayed graphically for the two treatment arms using error bars. Each pair of confidence intervals displayed for the two treatment arms observably overlapped.

The linear mixed models account for the correlational structure inherent in these repeated measures data, as intra-individual measures are more highly correlated than inter-individual measures. Since there was only one primary endpoint and only one endpoint was used to estimate the sample size, all statistical tests were conducted at the alpha = 0.05 level; no Bonferroni correction was employed. For the mixed model analyses, a linear model approach was used while allowing for the repeated measures to be generated for outcome assessment. The treatment effect was tested while controlling for the following covariates: time point, age, dehydration weight change, a gender-treatment-arm interaction effect, as well as baseline levels for systolic blood viscosity, diastolic blood viscosity, and plasma osmolality. Analyses of all outcome variables were performed using a mixed model, which takes into account intra-individual correlations across repeated measures.

## Results

One hundred adult participants completed the study. For each subject, the study required approximately 4–8 h of time on a single study date with no follow-up visits. Table 1 shows demographics of each study arm (CON versus ALK), including average age and the number of subjects by ethnicity were similar between the two study arms. Table 2 shows baseline characteristics for each study arm prior to exercise, including systolic and diastolic blood viscosities, hematocrit, plasma osmolality, bioelectric impedance analysis, body weight, systolic and diastolic blood pressures, heart rate, respiratory rate, and body temperature. The CON and ALK subjects did not differ significantly from baseline values.

**Table 1:** Demographics and baseline characteristics

Demographics	CON (*n* = 50)	ALK (*n* = 50)
Percent of Subjects by Gender		
Female	25%	25%
Male	25%	25%
Average Age in Years (SD)	31.96 (6.46)	30.36 (5.52)
Number of Subjects by Race/Ethnicity		
White	27	23
Black or African-American	14	20
Hispanic or Latino	4	5
Asian/Pacific Islander	5	2

**Table 2:** Baseline values for outcome measures (*n* = 100)

Variable	Mean	Std Dev	Min	Max
Systolic Blood Viscosity [millipoises]	38.5	4.3	30.9	54.5
Diastolic Blood Viscosity [millipoises]	110.6	17.5	76.6	170.8
Hematocrit [%]	43.1	3.1	37	50
Plasma Osmolality [mOsm/kg]	289.94	4.03	272	298
Bioelectrical Impedance Analysis				
Reactance Index	527	86	358	725
Capacitance Index	69	12	48	118
Impedance Index	532	86	362	729
Phase Angle	7.5	1.1	4.9	10.6
Total Body Water	39.2	8.7	25.1	60.9
Intracellular Water	22	5.5	14.3	34.5
Extracellular Water	17.2	3.4	10.8	26.3
Body Weight [kg]	72.1	14.47	46.2	105.8
Systolic Blood Pressure [mm Hg]	120	13	86	164
Diastolic Blood Pressure [mm Hg]	76	8	50	92
Heart Rate	65.8	11.3	37	93
Respiratory Rate	16.2	2	12	20
Body Temperature	97.8	0.8	95.1	99.5

The study involved between 4–8 h of time for each participant, depending upon the duration of the exercise period to achieve a dehydrated state. Study participants were monitored by a registered nurse from enrollment to discharge. There were no adverse events of any kind during the study. There were also no clinically significant abnormal values among the vital signs collected and laboratory evaluations performed. Systolic blood pressure, DBP, HR, respiratory rate, and body temperature were recorded at baseline, T000, and T120 min and are summarized in Table 3. Mean values for vital signs were similar in the two study arms. In addition, mean values with standard deviations for outcome parameters are also provided in Table 3.

**Table 3:** Results vs time

Hydration Markers		Baseline	T0	T15	T30	T45	T60	T75	T90	T120
SBV [millipoises]	CON	38.9 ± 3.9	41.1 ± 4.8	41.1 ± 4.9	41.3 ± 5.2		40.1 ± 4.9		39.3 ± 3.8	39.6 ± 4.7
	ALK	38.2 ± 4.6	40.5 ± 5.5	40.4 ± 5.8	39.7 ± 5.6		38.2 ± 4.4		38.0 ± 4.3	37.8 ± 4.4
DBV [millipoises]	CON	111.6 ± 16.9	120.5 ± 19.7	121.1 ± 19.4	119.9 ± 22.4		116.4 ± 20.5		113.7 ± 15.5	113.7 ± 20.7
	ALK	109.7 ± 18.1	121.4 ± 24.3	119.0 ± 22.6	115.4 ± 21.7		110.0 ± 18.5		108.3 ± 17.4	108.4 ± 17.8
OsmP [mOsm/kg]	CON	289.9 ± 4.3	295.8 ± 4.8	295.4 ± 4.93	291.8 ± 5.4		287.3 ± 5.1		286.2 ± 4.3	286.9 ± 3.7
	ALK	290.0 ± 3.8	294.9 ± 4.6	294.4 ± 4.6	290.9 ± 4.9		286.6 ± 4.7		285.3 ± 4.3	285.8 ± 3.6
BIA										
Reactance	CON	529.6 ± 84.1	526.5 ± 83.8			536.8 ± 84.7		542.6 ± 85.8		541.4 ± 90.0
	ALK	524.7 ± 87.9	511.8 ± 85.5			525.4 ± 92.4		528.2 ± 91.9		528.9 ± 97.5
Capacitance Index	CON	69.1 ± 12.3	66.4 ± 8.7			69.7 ± 8.9		70.8 ± 8.3		72.2 ± 12.4
	ALK	68.4 ± 11.5	66.6 ± 12.4			68.4 ± 8.4		69.2 ± 9.1		69.9 ± 9.1
Impedance Index	CON	534.4 ± 84.8	530.9 ± 83.9			541.4 ± 84.8		547.4 ± 86.0		537.6 ± 114.8
	ALK	529.2 ± 88.0	517.6 ± 85.9			530.5 ± 92.2		533.4 ± 91.6		533.9 ± 87.3
Phase Angle	CON	7.5 ± 1.0	7.3 ± 1.0			7.5 ± 1.0		7.5 ± 0.9		7.7 ± 1.4
	ALK	7.5 ± 1.3	7.4 ± 1.1			7.5 ± 1.1		7.6 ± 1.1		7.6 ± 1.1
TBW	CON	38.6 ± 8.8	38.1 ± 8.6			38.1 ± 8.6		37.8 ± 8.4		38.0 ± 8.7
	ALK	39.8 ± 8.6	40.3 ± 8.6			39.8 ± 8.6		39.7 ± 8.5		39.5 ± 8.3
ICW	CON	21.7 ± 5.5	21.6 ± 5.4			21.5 ± 5.4		21.4 ± 5.3		21.5 ± 5.4
	ALK	22.3 ± 5.5	22.6 ± 5.5			22.3 ± 5.5		22.3 ± 5.5		22.3 ± 5.3
ECW	CON	16.8 ± 3.5	16.8 ± 3.4			16.6 ± 3.4		16.4 ± 3.3		16.5 ± 3.4
	ALK	17.5 ± 3.4	17.7 ± 3.3			17.5 ± 3.3		17.4 ± 3.3		17.3 ± 3.2
Body Weight [kg]	CON	70.7 ± 13.8	69.1 ± 13.5			70.4 ± 13.7		70.2 ± 13.7		70.2 ± 13.8
	ALK	73.5 ± 15.1	71.9 ± 14.7			73.1 ± 15.0		73.2 ± 15.0		73.0 ± 15.0
Vital Signs										
SBP [mm Hg]	CON	118.9 ± 12.0	112.4 ± 11.9							115.1 ± 12.3
	ALK	121.9 ± 13.4	114.5 ± 10.7							115.8 ± 13.9
DBP [mm Hg]	CON	75 ± 8.8	75.1 ± 7.3							75.7 ± 8.8
	ALK	77.4 ± 7.6	73.0 ± 8.5							75.0 ± 8.5
HR [bpm]	CON	66.1 ± 11.5	83.6 ± 15.5							69.9 ± 11.7
	ALK	65.5 ± 11.2	84.8 ± 12.7							70.4 ± 12.8
Respiratory Rate [bpm]	CON	16.2 ± 2.0	17.4 ± 2.1							16.6 ± 1.8
	ALK	16.1 ± 2.2	17.3 ± 1.6							17.2 ± 1.8
Body Temperature [°C]	CON	97.8 ± 0.9	98.7 ± 0.5							97.9 ± 0.6
	ALK	97.7 ± 0.6	98.6 ± 0.6							97.9 ± 0.5

*CW* control water, *AW* alkaline water, *SBV* systolic blood viscosity, *DBV* diastolic blood viscosity, *Hct* hematocrit, *OsmP* plasma osmolality, *BIA* bioelectrical impedance, *TBW* total body water, *ICW* intracellular water, *ECW* extracellular water, *SBP*, systolic blood pressure, *DBP* diastolic blood pressure, *HR* heart rate

The percentage change during the rehydration period from T000 to T120 min was computed for each outcome measure, reflecting the overall magnitude of hydration during the rehydration and recovery period, following standardized exercise-induced dehydration, while compensating for inter-individual differences at baseline and T000 min. Subjects acted as their own controls, and the inter-individual variability of endpoints was moderated by dividing the difference between the subject’s dehydrated state (T000 min) and final rehydrated state (T120 min) by the value of each subject’s own dehydrated state (T000 min).

After rehydration and recovery, the average percentage change for systolic blood viscosity, measured at a high-shear rate of 300 s^-1^, in subjects administered CON was 3.36%; whereas for ALK, the average percent change was 6.30% (*p* = 0.03). Nominally, ALK significantly reduced and restored high-shear blood viscosity during a 120-min rehydration period by 87.50% more than CON. After rehydration and recovery, the average percentage change for diastolic blood viscosity (measured at low-shear rate: 5 s^-1^) in subjects administered CON was 5.43%, while the mean percent change for ALK was 9.35%. Furthermore, no other outcome variables, serving as hydration markers, demonstrated a significant difference between the two treatment arms when comparing the percent change in the outcome measure during the rehydration period (T000 to T120 min, Table 4).

**Table 4:** Average percent change during rehydration (T000 vs. T120 min)

Endpoint	CON (*n* = 50)	ALK (*n* = 50)	*p* value
Systolic Blood Viscosity	3.36 [1.46, 5.26]	6.30 [4.51, 8.09]	0.026
Diastolic Blood Viscosity	5.43 [2.41, 8.44]	9.35 [6.19, 12.50]	0.074
Plasma Osmolality	3.01 [2.72, 3.29]	3.07 [2.78, 3.36]	0.751
Bioimpedance Analysis			
Reactance	-2.85 [-4.44, -1.27]	-3.45 [-4.36, -2.53]	0.514
Capacitance	-8.92 [-12.78, -5.06]	-6.09 [-8.96, -3.21]	0.240
Impedance	-1.23 [-5.26, 2.81]	-3.27 [-4.34, -2.21]	0.329
Phase Angle	-6.09 [-11.44, -0.74]	-3.43 [-5.67, -1.19]	0.360
Total Body Water	1.25 [0.17, 2.33]	1.86 [1.26, 2.47]	0.325
Intracellular Water	0.47 [-0.73, 1.68]	1.32 [0.79, 1.85]	0.200
Extracellular Water	2.20 [1.15, 3.25]	2.49 [1.63, 3.36]	0.663
Weight [kg]	-1.59 [-1.76, -1.42]	-1.59 [-1.74, -1.43]	0.963

Above data are mean values for percentage differences [95% confidence intervals]

Further analyses, using PROC REG in SAS provided an estimate of the best fitting line per treatment arm, as well as the slope and intercept parameters. The period of rehydration from T000 to T120 min was used to determine the best-fit regression line for each arm and endpoint. No significant difference was detected in the slope parameter between the two treatment arms for each endpoint. This analysis of slopes was used to examine the rate of change for each endpoint parameter during the rehydration period (see Table 5). A significant difference between the two treatment arms would reflect a faster hydration rate. A trend was observed for mean systolic and diastolic blood viscosity levels, which decreased faster (greater negative slope) for ALK as compared with CON. Impedance, an index derived from bioelectrical impedance analysis, was observed to increase faster (greater positive slope) for ALK as compared with CON.

**Table 5:** Slope analyses for serial measurements of outcome parameters

	Linear Regression Slopes			Curvilinear Regression
Endpoint	CON (*n* = 50)	ALK (*n* = 50)	*p* value	*p* value
Systolic Blood Viscosity	-0.017	-0.026	0.356	0.555
Diastolic Blood Viscosity	-0.071	-0.114	0.261	0.364
Plasma Osmolality	-0.086	-0.087	0.911	0.839
Bioimpedance Analysis				
Reactance	0.128	0.140	0.951	0.967
Capacitance	0.048	0.028	0.374	0.830
Impedance	0.064	0.133	0.741	0.828
Phase Angle	0.003	0.002	0.605	0.978
Total Body Water	-0.004	-0.006	0.912	0.969
Intracellular Water	-0.001	-0.003	0.894	0.985
Extracellular Water	-0.014	-0.004	0.944	0.940
Weight	0.008	0.009	0.988	0.995

Figure 2 shows systolic blood viscosity changes as a function of time, where the 2 treatment groups had similar viscosity levels at baseline. The parallel slopes for the 2 study arms measured from baseline to T000 min (i.e., end of the exercise period and the beginning of the rehydration period) suggests both study arms achieved a similar rate of dehydration during exercise. After T000, when the subjects began ingestion of water, a steeper slope can be observed for the ALK group than for CON group, demonstrating an enhancement in the recovery period towards restoring pre-exercise baseline levels. By T060 min, midway through the recovery period, mean systolic viscosity levels for ALK subjects returned to the pre-exercise baseline levels, whereas the CON did not return to pre-exercise baseline levels even at T120 min. This pattern is observed visually in the graphic display and consistent with the comparison of the percent changes in systolic viscosity. However, these noted differences that were significant using a comparison of percent changes from T000 to T120 min could not be detected using absolute differences based on 95% confidence intervals, as shown in Fig. 2, probably due to the large inter-individual variability.

Similar results were observed for diastolic blood viscosity as shown in Fig. 3. The values at baseline were even closer for the two groups. The increases found with exercise, between baseline and T000, progressed at a similar rate for both treatment groups. Based on mean levels for diastolic viscosity, Fig. 3 shows a more pronounced rehydration rate for ALK than CON with failure to return to baseline levels for mean diastolic viscosity in the CON group by T120 min.

Using mixed model analyses, the treatment effect of ALK vs. CON was observed to be significant for systolic blood viscosity (*p* = 0.02). The treatment effect of ALK vs. CON was not observed to be significant for the other outcome measures of diastolic blood viscosity, plasma osmolality, or the bioelectrical impedance indices. The mixed model analysis appeared to confirm the significant difference in the effect of ALK on blood viscosity, showing that after controlling for the effect of multiple covariates using a mixed model, ALK had a statistically significant effect on systolic blood viscosity when compared with CON. When the analysis was repeated with the interaction of treatment-effect-by-time included as a variable in the mixed model, the treatment effect was still significant for systolic blood viscosity (*p* = 0.02) in favor of ALK; however, the interaction effect of treatment-arm-by-time-point for systolic blood viscosity was not itself significant.

## Discussion

This randomized, double-blinded, parallel-arm controlled study compared the rehydration effect of ALK to CON in order to characterize relative hydration efficacy and performance. A pre-planned analysis of percentage changes, starting at dehydration (T000) and ending at recovery (T120), enabled the two treatment groups to be compared while reducing the impact of inter-individual variability. For systolic blood viscosity, ALK demonstrated significantly greater rehydration than CON (*p* = 0.03), and this result was consistent with the findings using the mixed model analyses.

Interest in the study of biomarkers for hydration has intensified in recent years, however the relative utility of markers is dependent on the environment and the nature of the stimuli applied in a given study. Even in studies of responses to acute exercise-induced dehydration, a gold standard biomarker for hydration status has proved elusive [13–15]. Viscosity was used as the primary endpoint in this study to reflect intravascular hydration and was clearly affected by exercise-induced dehydration. Several prior studies have reported increases in blood viscosity following exercise [16, 17]. In a study of 20 healthy adults, blood viscosity was reported to increase after 15 min of submaximal exercise [18]. In a prior clinical study of 47 endurance-trained and untrained females, mean viscosity levels after 1 h of maximal exercise were reported to be 12.6% higher, a greater magnitude increase than could be attributed to hematocrit, which rose by a mean of 8.9% [19]. Blood viscosity is not static but changes dramatically depending on shear rate. Shear rate is calculated by dividing flow velocity by lumen diameter. When blood moves quickly at the peak of systole, it is at high-shear and relatively thinner because erythrocytes are dispersed. At high shear rates, systolic viscosity is influenced by hematocrit levels and red cell deformability, whereas at low shear rates, diastolic viscosity is influenced by red cell aggregation [20]. For this reason, systolic blood viscosity may be able to provide a more direct marker of hydration status than diastolic blood viscosity.

The key difference between electrolyzed, high-pH ALK and standard drinking water purified by reverse osmosis, used as the CON in this study is the degree of alkalinity. In a study of 1136 Japanese females, Murakami et al. found acidic dietary load was independently associated with significantly increased SBP and DBP, low density lipoprotein (LDL) and total cholesterol levels, BMI, and waist circumference [21]. These researchers suggested that unfavorable metabolic cardiac risk factors may be induced by mild metabolic acidosis which increased cortisol production. Heil reported significantly increased blood pH secondary to consumption of mineral-rich ALK [22]. Separately, Heil et al. demonstrated faster and better overall hydration with ALK than CON (bottled) in ten male cyclists. Hydration markers reported therein were urine specific gravity, urine output, serum protein concentration, and water retention [23]. In both of these studies, the effects took at least one week to occur after habitual intake of alkaline water. While Heil et al. did not perform mechanistic studies, they hypothesized that blood alkalinity was shifted as a result of direct absorption of alkaline minerals into the blood and that water retention within the vasculature was improved by the absorption of additional minerals into the blood [22]. In a more recent study by the same group, it was suggested that increases in extracellular pH may influence blood flow indirectly by altering interstitial potassium concentrations [24].

Separately, a study using an exercise-induced dehydration protocol to compare the effect of two fluid replacement beverages on markers for oxidative stress showed that rehydration recovery following ingestion of either a carbohydrate-electrolyte beverage or water reduced levels of malondialdehyde, a common marker for oxidative stress, relative to plasma concentrations of malondialdehyde at a dehydrated state [25]. Disruptions in blood flow promote an oxidative state where reactive oxygen species accumulate. Red blood cells in particular are vulnerable to an oxidative environment in the human body and, as a consequence of their iron content, are capable of producing their own free radicals [26]. This process of autoxidation occurs when oxygenated hemoglobin is degraded and releases a superoxide. Concurrently, the ferrous (Fe^2+^) state iron in hemoglobin is oxidized to ferric (Fe^3+^) hemoglobin, producing methemoglobin which is incapable of transporting oxygen [27]. Peroxides in the body degrade hemoglobin proteins and cause erythrocytes to release heme and iron. Forces required for red cells to perfuse capillaries can cause cell membranes to leak ions, causing further damage to lipid membranes [28]. When reactive oxygen species initiate peroxidation of lipid membranes, cellular membrane proteins often become cross-linked and red cells become stiffer with less deformability [27]. Production of methemoglobin, modification and degradation of proteins, cross-linking of membrane proteins, lipid peroxidation, hemoglobin cross-linking, and impaired surface properties are all mechanisms by which oxidative stress functionally modifies red blood cells [26]. These mechanisms alter red cell properties, including reduced membrane fluidity and increased aggregation, leading to increased blood viscosity and impaired flow [29].

A separate study of 154 subjects with varying stages of diabetes mellitus and healthy controls showed that more than 76% of oxidative stress in apparently healthy subjects was associated with elevated WBV, with 95% prevalence in the prediabetes group and 92% prevalence in the diabetes group [30]. This clinical study measured markers of erythrocyte oxidative stress included erythrocyte glutathione, methemoglobin, and malondialdehyde. Associations between oxidative stress of red blood cells and altered blood viscosity in healthy subjects, as well as those with diabetes and prediabetic patients, suggest that blood viscosity may be a marker for underlying oxidative stress.

We speculate that differences in systolic viscosity levels caused by ALK vs. CON following dehydration may have been mediated by the influence of reactive oxygen species on erythrocyte membranes and their deformability. Further studies are needed to determine if high-pH ALK is directly associated to reductions in oxidative stress. With respect to plasma osmolality as a hydration marker, Armstrong in his authoritative review noted that “a single gold standard, including plasma osmolality, is not possible for all hydration assessment requirements” [15]. He stated body mass change is the most accurate assessment of hydration in real time, and his review of biomarkers, which did not include blood viscosity, suggested that the accuracy of most hydration markers is not consistently supported. Body mass changes reflect body water losses and gains secondary to sweating and water intake, respectively. Consequently, changes in mass are very frequently measured in exercise studies and serve as a benchmark for other hydration markers. Although plasma osmolality is considered among the best available indices by many researchers, none of the analyses performed in this study showed significant differences between ALK and CON on this marker. Plasma osmolality does not incorporate the influence of cellular content in the blood and is difficult to assess when total body water, fluid intake, and fluid loss are altered.

Bioelectrical impedance analysis has been widely used to assess hydration status. This tool allows for the determination of water volumes throughout various fluid compartments of the body. There were no treatment arm effects when comparing ALK with CON on any of the bioimpedance indices in our study. It is possible that acute dehydration and rehydration consistent within this present study (2% body mass) failed to accurately predict changes in body water that were otherwise able to be determined by assessing body mass changes. Further, in athletes with low baseline body fat, small body water changes may be mistakenly reported as body fat changes by bioimpedance testing [31]. Changes in extracellular volume and osmolality may also impair the accuracy of bioelectrical impedance assessments [32].

## Conclusion

This study was designed to characterize differences between ALK and CON in terms of intravascular hydration as quantified by serial changes in systolic blood viscosity following exercise-induced dehydration. Drinking high-pH ALK was shown to reduce systolic blood viscosity significantly more than CON consumption following exercise-induced dehydration, when comparing the percent change in WBV from a dehydrated state to 120 min after rehydration and recovery. A mixed model analyses validated this significant treatment effect for high-pH ALK on systolic blood viscosity vs. CON. Absolute differences at multiple time points did not demonstrate any significant differences; however the subjective observed benefit may be attributed to the high variability of WBV measurements in the study groups.

## Abbreviations

ALK: Alkaline water
BMI: Body mass index
CON: Control
DBP: Diastolic blood pressure
HR: Heart rate
LDL: Low density lipoprotein
PROC MIXED: Mixed model procedure
PROC REG: Regression procedure
SAS: Statistical analysis system
SBP: Systolic blood pressure
WBV: Whole blood viscosity.
